# Consequences of the crosstalk between monocytes/macrophages and natural killer cells

**DOI:** 10.3389/fimmu.2012.00403

**Published:** 2013-01-04

**Authors:** Tatiana Michel, François Hentges, Jacques Zimmer

**Affiliations:** Laboratory of Immunogenetics and Allergology, Centre de Recherche Public de la SantéLuxembourg, Luxembourg

**Keywords:** NK cells, macrophages, monocytes, receptors, cytokines, activation, infection

## Abstract

The interaction between natural killer (NK) cells and different other immune cells like T cells and dendritic cells is well-described, but the crosstalk with monocytes or macrophages and the nature of ligands/receptors implicated are just emerging. The macrophage-NK interaction is a major first-line defense against pathogens (bacteria, viruses, fungi, and parasites). The recruitment and the activation of NK cells to perform cytotoxicity or produce cytokines at the sites of inflammation are important to fight infections. The two main mechanisms by which macrophages can prime NK cells are (1) activation through soluble mediators such as IL-12, IL-18, and (2) stimulation through direct cell-to-cell contact. We will discuss the progress in matters of modulation of NK cell functions by monocytes and macrophages, in the steady state and during diseases.

## Introduction

Resident macrophages derive from circulating monocytes. Their differentiation occurs upon entering the tissues where macrophages show heterogeneous phenotypes depending on local microenvironments (Taylor et al., 2005). Macrophages are found in lymphoid as well in non-lymphoid organs like liver (Kuppfer cells), lung (alveolar macrophages), nervous system (microglia), reproductive organs, and in the lamina propria of the gut (Taylor et al., 2005; Richards et al., 2012). When pathogens infect specific organs, one of the earliest cell types to arrive at the site of infection is natural killer (NK) cells in partnership with macrophages. Important differences have been shown among the molecular mechanisms for NK cell activation by microorganisms (including viruses, bacteria, and protozoans) involving inhibitory and activating receptors (Newman and Riley, 2007; Horowitz et al., 2011). The recognition and lysis of target cells by NK cells pass through sensing “missing self” due to the lack of MHC class I molecules and through interaction of activating receptors with specific ligands.

How does the interaction between monocytes or macrophages and NK cells trigger immune response? Both cell to cell contact and soluble mediators contribute to the activation of NK cells. A new understanding of NK cell responses in health and in infectious diseases is emerging where macrophages play a crucial intermediary role. Here we summarize recent data regarding the molecular interactions that regulate the monocyte/macrophage-dependent activation of NK cells.

## Macrophage/NK interactions by cell-to-cell contact: which receptors are implicated?

In human and mouse models, a simple coculture of monocytes or macrophages with NK cells increases CD69 expression in NK cells, showing that the former can activate NK cells *in vitro* (Haller et al., 2002; Dalbeth et al., 2004; Scott et al., 2004). This activation is cell-contact-dependent and enhances the secretion of IFN-γ by NK cells (Haller et al., 2002; Atochina and Harn, 2005). Several groups have shown an upregulation of CD69 at the NK cell surface, the production of IFN-γ and the degranulation of NK cells when monocytes/macrophages were previously stimulated by molecules like Lacto-N-fucopentaose III, poly I:C, CpG DNA, and LPS (Chace et al., 1997; Scott et al., 2004; Atochina and Harn, 2005; Basu et al., 2009; Bellora et al., 2010; Zhou et al., 2012). Infection by parasites, like *P. falciparum* and *Leishmania* (Aranha et al., 2005; Baratin et al., 2005), or viruses, like influenza A virus, Sendai virus, human cytomegalovirus (Siren et al., 2004; Romo et al., 2011), or bacteria like *Salmonella, M*. *tuberculosis, E. faecalis, S. aureus, Lactobacillus, S. pneumonia*, and *B. anthracis*, has the same effects (Brill et al., 2001; Haller et al., 2002; Schierloh et al., 2005; Denis et al., 2007; Newman and Riley, 2007; Lapaque et al., 2009; Takayama et al., 2010; Elhaik-Goldman et al., 2011; Klezovich-Benard et al., 2012; Souza-Fonseca-Guimaraes et al., 2012).

The dialog between monocytes or macrophages and NK cells via cellular contacts enhances NK cell activity. Depending on the pathogen, different types of receptors present at the NK cell surface respond to the stimulated macrophages (Table 1).

**Table 1:** **Ligand-receptor families implicated in activation of NK cells by monocytes/macrophages**.

**Stimulated with**	**Monocyte/macrophage ligands**	**NK receptors**	**Species**	**Selected références**
Various pathogens		CD69	Human, mouse	Scott et al., 2004; Haller et al., 2002; Dalbeth et al., 2004
LPS, Lacto-N fucopentaose III	CD40	CD154	Mouse	Scott et al., 2004; Atochina and Harn, 2005
Cytomegalovirus	CD48	2B4	Human	Bellora et al., 2010; Romo et al., 2011; Nedvetzki et al., 2007
LPS, *M. Tuberculosis* poly I:C	ULBP1-3 MICA	NKG2D	Human, mouse	Nedvetzki et al., 2007; Kloss et al., 2008; Hamerman et al., 2004; Basu et al., 2009; Hou et al., 2009; Vankayalapati et al., 2005
Influenza A virus, Sendai virus	MICB	NKG2D	Human	Siren et al., 2004
LPS, lipopeptide Pam3CSK4, zymosan, poly I:C, *B. anthracis* spores	RAE-1	NKG2D	Mouse	Hamerman et al., 2004; Klezovich-Benard et al., 2012
*S. pneumoniae*, cytomegalovirus, *M. tuberculosis*		NKp46	Human, mouse	Hou et al., 2009; Elhaik-Goldman et al., 2011; Romo et al., 2011
		NKp30, NKp44	Human	Nedvetzki et al., 2007
LPS, lipopeptide Pam3CSK4, poly l:C	AICL	NKp80	Human	Welte et al., 2006
Poly l:C	Qa-1	NKG2A	Mouse	Zhou et al., 2012

### CD154-CD40 interactions

In mice, the CD40-CD40L (CD154) interactions participate in driving the activation of NK cells by LPS- or Lacto-N-fucopentaose III-activated macrophages *in vitro* (Table 1) (Scott et al., 2004; Atochina and Harn, 2005).

### NKG2D interactions

In human, the presence of high doses of LPS changes the phenotype of macrophages by inducing the expression of various ligands of the activating receptor NKG2D: UL16-binding proteins (ULBP1, ULBP2, and ULBP3) and MHC class I-related chain A (MICA) (Nedvetzki et al., 2007). Human NK cells which are in contact with macrophages or LPS-activated macrophages express increased levels of NKG2D. However, NKG2D expression in NK cells seems to be less induced by LPS-activated macrophages than unactivated macrophages (Nedvetzki et al., 2007). This phenomenon could be similar to the observation that prolonged exposure to its ligands induces a downregulation of NKG2D on NK cells (Ogasawara et al., 2003; Hamerman et al., 2004; Coudert et al., 2005; Kloss et al., 2008; Lapaque et al., 2009). These receptor-ligand interactions induce the lysis of macrophages stimulated with high doses of LPS, by NK cells via NKG2D (Nedvetzki et al., 2007). Thus, NK cells specifically kill PBMC-derived macrophages stimulated with high doses of LPS probably to eliminate overstimulated macrophages to avoid endotoxic shock. On the contrary, LPS-stimulated microglia (nerve system macrophages) is less susceptible to NK cell-mediated cytotoxicity compared to resting microglia. The killing of resting microglial cells by NK cells is reduced by NKG2D-specific blocking Abs, meaning that NKG2D is involved in this process. Furthermore, NKG2D ligands are expressed constitutively by microglia cells *in vitro*. However, in LPS-stimulated microglia this expression is down-regulated. By this mechanism, activated microglial cells which are not lysed by NK cells could be able to present antigens to infiltrating T cells and initiate a limited immune response in the brain (Lunemann et al., 2008). Another study on monocytes described that the killing of MICA-expressing monocytes by autologous NK cells is not observed neither in the presence nor in the absence of LPS (Kloss et al., 2008). In presence of LPS, MICA is up-regulated on monocytes. Differences between these various cell types show that macrophages, microglia, and monocytes are not equally susceptible to NK cell-mediated killing after LPS-stimulation. This could be the result of a variation in the level of inhibitory and activating signals from these target cells to NK cells. This might be explained by the difference of NKG2D engagement and different levels of the counterbalance due to MHC class I binding of inhibitory NK cell receptors. Indeed, in presence of LPS, PBMC-derived macrophages are not associated with an MHC class I up-regulation while this is the case for microglia and monocytes (Nedvetzki et al., 2007; Kloss et al., 2008; Lunemann et al., 2008). NKG2D is also required in other organs but the nature of its ligand differs depending on the type of infection. Thus, poly I:C stimulated uterine macrophages up-regulate MICA and activate IFNγ production of uterine NK cells through the recognition of NKG2D (Basu et al., 2009). Likewise, alveolar macrophages infected by *M. tuberculosis* are lysed by NK cells via the interaction between NKG2D and ULBP1 (Vankayalapati et al., 2005). Another NKG2D ligand, MICB, is expressed when human macrophages are infected with influenza A or Sendai viruses and the infected macrophages are likely to promote IFN-γ release by NK cells (Siren et al., 2004).

In mice, similar pathways can be found to initiate the activation of NK cells. Thus, peritoneal macrophages treated with poly I:C increase NKG2D expression in NK cells (Zhou et al., 2012). In response to Toll-like receptor (TLR) ligands like LPS, lipopeptide Pam_3_CSK_4_, zymosan, or poly I:C, retinoic acid early inducible-1 (RAE-1) is induced in macrophages (Hamerman et al., 2004). After contact with RAE-1, the NKG2D receptor triggers activation of NK cell cytotoxicity-related molecules, TNF-related apoptosis-inducing ligand (TRAIL), perforin and FasL and therefore the induction of NK cell cytotoxicity toward tumor cells and the secretion of IFN-γ (Hou et al., 2009; Zhou et al., 2012). RAE-1-NKG2D interactions are also involved to stimulate the release of IFN-γ by NK cells during the crosstalk with monocytic myeloid derived suppressor cells (MDSC) (Nausch et al., 2008). The same RAE-1-NKG2D engagement is observed between bone marrow-derived macrophages and NK cells in response to *B. anthracis* spores (Klezovich-Benard et al., 2012). Thus, the NKG2D pathway has its importance to confer protection against infections, as it indirectly detects a wide variety of pathogens through the recognition of various NKG2D ligands.

### 2B4-CD48 interactions

In human, the release of IFN-γ by NK cells can be triggered by the interaction of the receptor 2B4 with the ligand CD48 present at the surface of PBMC-derived macrophages (Nedvetzki et al., 2007; Bellora et al., 2010; Romo et al., 2011). This engagement depends on how macrophages have been prearmed. Macrophages stimulated with high doses of LPS activate NK cytotoxicity rather than cytokine release. When the latter have not been activated or activated with low doses of LPS, the system passes through the 2B4-CD48 pathway. The 2B4-CD48 engagement has other consequences like the increase of the expression of 2B4 and the proliferation of NK cells (Nedvetzki et al., 2007). Thus, after contact with macrophages, 2B4 induces NK cell cytokine secretion and proliferation. However, depending on the cell types, the signaling triggered by 2B4 could also result in activation of NK cytotoxicity, which could be due to a different 2B4 isoform expression (Mathew et al., 2009; Kim et al., 2010). When macrophages are infected by human cytomegalovirus, the expression of CD48 is down-regulated, suggesting the existence of a potential viral immune escape strategy targeting the 2B4 pathway (Romo et al., 2011).

### Immune synapses

One group has investigated the structure of the immune synapses between macrophages and NK cells in the context of LPS-stimulation (Nedvetzki et al., 2007). Images of the macrophage-NK cell conjugates show an accumulation of F-actin at the site of contact which is more frequent when macrophages are stimulated with high doses of LPS. The accumulation of F-actin at cytolytic synapses between NK cells and LPS-stimulated macrophages happens at the NK cell side, while F-actin accumulates at the macrophage side in the non-cytolytic synapses in absence of LPS. Thus, the NK cell-macrophage communication is different depending on whether macrophages are treated or not with high doses of LPS. Indeed, the activating receptor complex NKG2D/DAP10, the activating adaptor protein CD3ζ as well as ICAM-1 are recruited to the NK cell immune synapse by macrophages stimulated with high doses of LPS and very rarely by unactivated macrophages, showing a key role of these molecules in triggering cytotoxicity. On the other hand, a large fraction of 2B4 clustered to the immune synapse of macrophages and macrophages activated with low doses of LPS, confirming the concept that this receptor is important for the macrophage-mediated NK cell proliferation and IFN-γ secretion. Two distinct NK cell synapses are defined, one activating the proliferation and cytokine secretion, by the recruitment of 2B4, and the other one triggering lysis through the activation of NKG2D (Nedvetzki et al., 2007).

### NKP46-DNAM-1 interactions

In human, the susceptibility of macrophages to be lysed by autologous NK cells can also be mediated by the activating receptors NKp46 and DNAM-1. Thus, blocking of NKp46 and DNAM-1 decreases NK cell cytotoxicity against cytomegalovirus-infected macrophages and LPS-stimulated macrophages derived from PBMC. NKp46 is also involved in the lysis of *M*. *tuberculosis*-infected alveolar macrophages (Vankayalapati et al., 2005; Bellora et al., 2010; Romo et al., 2011). In the case of resting microglial cells, only NKp46 engagement is implicated in the killing (Lunemann et al., 2008). Furthermore, the coculture of NK cells and PBMC-derived macrophages increases the expression of NK activating receptors like NKp30, NKp44, and NKp46 (Nedvetzki et al., 2007). In regard of the different data reported, it seems that NKp46 participates mainly to NK cell cytolytic activity whereas 2B4 is rather involved in the induction of IFN-γ production. DNAM-1 plays quite a dual role, as it seems to participate to the activation of both cytotoxicity and IFN-γ secretion.

In contrast to human, the expression of mouse NKp46 and DNAM-1 is not increased in the presence of peritoneal macrophages treated with poly I:C (Zhou et al., 2012). However, in another context, NCR1 (mouse NKp46) is shown to be involved in the macrophage-NK cell crosstalk, as alveolar macrophages infected *in vitro* by *S. pneumoniae* mediate lung NK cell activation (CD107a expression and IFN-γ secretion) in part through the NCR1 receptor pathway (Elhaik-Goldman et al., 2011).

Other molecules, like CD44 and ICAM-1, could be involved in macrophage-NK cell interactions, but their precise role remains to be determined (Nedvetzki et al., 2007).

### NKp80-AICL interactions

In human, PBMC-derived macrophages and monocytes express the activation-induced C-type lectin (AICL). Monocytes activated through their TLR by LPS, lipopeptide Pam_3_CSK_4_, or poly I:C increase the expression of AICL at their surface. AICL is the ligand of the NK cell activating receptor NKp80. The interaction AICL-NKp80 leads to the secretion of IFN-γ by NK cells (Welte et al., 2006). TLR-induced AICL expression may act to eliminate monocytes or macrophages exposed to or infected by microorganisms.

### NKG2A-Qa-1 interactions

Both activating and inhibitory surface receptors participate in the regulation of NK cell activities. Thus, Qa-1, the ligand of the inhibitory receptor NKG2A is upregulated at the surface of poly I:C stimulated-macrophages. However, the NKG2A level does not change when NK cells are cocultured with these macrophages. The Qa-1-NKG2A interactions allow the macrophages to escape to lysis by NK cells (Zhou et al., 2012).

## Macrophage/NK interactions through soluble factors

The first observations of cytokines being secreted by macrophages and influencing NK cell functions are from the early 1990s (Lemaire and St-Jean, 1990; Tripp et al., 1993; Lauzon and Lemaire, 1994). In 1993, Tripp et al. observed that *Listeria monocytogenes* is phagocytosed by peritoneal macrophages, which then release TNF-α and IL-12 resulting in the production of IFN-γ by NK cells (Tripp et al., 1993). The following year, another team showed that alveolar macrophages inhibited lung NK cell activities through the release of TGF-β 1 (Lauzon and Lemaire, 1994).

### IL-12, IL-18 messengers

More recently, the proinflammatory cytokines IL-6, IL-12, IL-18, and TNF-α were observed in cultures of mouse spleen NK cells and peritoneal macrophages (Scott et al., 2004). The cytokine production between single cell-type cultures and cocultures showed no differences except a decrease of the IL-6 secretion by macrophages when they were in contact with NK cells. However, the inhibition of IL-12 secretion from activated macrophages has no influence on NK cell function during bacterial peritonitis (Scott et al., 2004). Thus, the potential activators IL-12 and IL-18 do not play a major role in NK cell activation by macrophages under these conditions. On the contrary, the secretion of IL-12 by CpG DNA or *B. anthracis*-infected macrophages enhances the release of IFN-γ by mouse NK cells but needs in addition a cell-to-cell contact (Chace et al., 1997; Klezovich-Benard et al., 2012). Moreover, IL-12 and IL-18 are involved in the crosstalk between Kupffer cells (liver resident macrophages) and NK cells after poly I:C treatment in mice (Hou et al., 2009).

**Figure 1:**
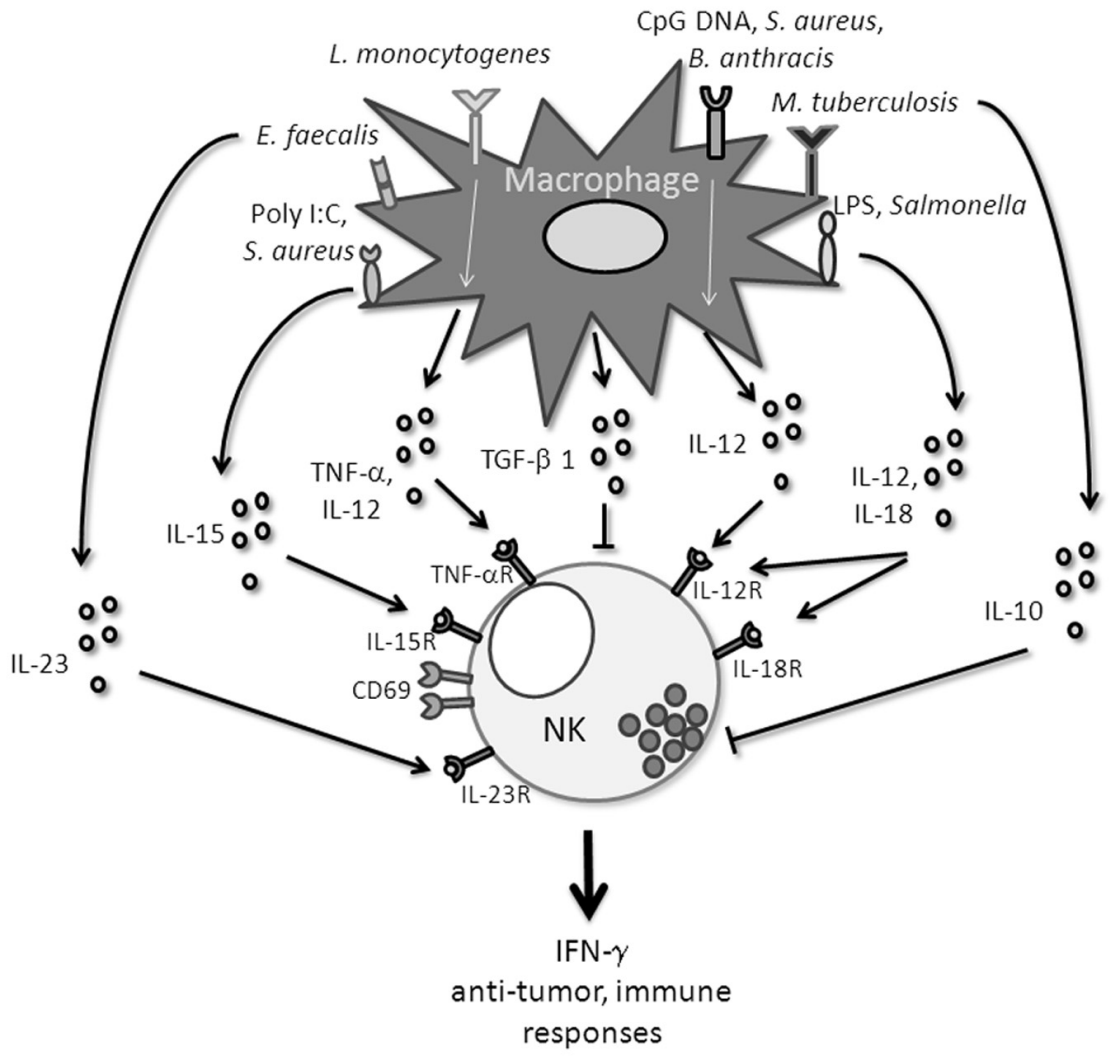
**Different macrophage-derivated signals that influence NK cell functions**.

The same capacity of IL-12 and IL-18 secreted by Kupffer cells to activate IFN-γ production by liver NK cells is found in human (Tu et al., 2008). The key role of IL-12 and IL-18 in NK cytotoxicity is confirmed in an experiment where PBMC-derived macrophages are infected with *Salmonella* (Lapaque et al., 2009). Furthermore, LPS, in presence of PBMC-derived macrophages, promotes the expression of IL-18 and consequently the synthesis of IFN-γ by NK cells (Bellora et al., 2012). Finally, *S. aureus* stimulated-monocytes from PBMC, produce IL-12 which participates to IFN-γ secretion by NK cells (Figure 1) (Haller et al., 2002).

### IL-15 messenger

On the other hand, the release of the proinflammatory cytokine IL-15 by mouse poly I:C-stimulated macrophages, also promotes the production of IFN-γ and the expression of CD69 by NK cells (Zhou et al., 2012). Furthermore, macrophages isolated from lungs infected by *S. aureus* represent a source of IL-15 capable to induce TNF-α production by NK cells (Small et al., 2008). Another team has recently shown that mice infected by *L. monocytogenes* have inflammatory monocytes and subsets of spleen macrophages which mediate granzyme B and IFN-γ secretion by NK cells *in vivo* (Soudja et al., 2012). After detection of different classes of microbial pathogens, activated monocytes and macrophages upregulate and *trans*-present IL-15 to NK cells.

### IL-23 messenger

Recently, a study described that IFN-γ–producing NKp46^+^ NK cells were increased in the intestine of patients with Crohn's disease. The production of IFN-γ by these cells is stimulated by the secretion of IL-23 and by cell-to-cell contact with the CD14^+^ lamina propria macrophages in response to stimulation with *E. faecalis* (Takayama et al., 2010). IL-23 may be involved as a key mediator in gut-specific inflammation.

### IL-10 messenger

Monocytes have also the capacity of a net inhibitory effect on NK cell activation through the release of IL-10 after an infection with *M. tuberculosis* and a depletion of these monocytes will increase the secretion of IFN-γ by NK cells (Schierloh et al., 2005).

The cytokines released by monocytes and macrophages to mobilize NK cells require the recognition of “danger” molecules by innate immune receptors. Then the triggering of pathways which involve NK-cell mediated IFN-γ release will promote the expression of antimicrobial functions of effector cells to protect from pathogens (Figure 1).

### Influence of the tissue environment

The outcome of the interaction between macrophages and NK cells is depending on their tissue localization (Zoller and Matzku, 1982; Raz, 2007). Indeed macrophages derived from PBMC have not the same phenotype than macrophages localized in liver. There is lower expression of HLA class I and greater expression of HLA class II molecules on Kupffer cells (Tu et al., 2008). The same phenomenon is observed for NK cells. Indeed NK cells from the liver are phenotypically different from blood NK cells due to an absence of CD16 expression (Tu et al., 2008). Mouse NK cells from lung and spleen are quite different and react differently in contact with spleen or lung macrophages. Spleen macrophages have the tendency to induce a stronger activation of NK cell cytotoxicity than lung macrophages (Michel et al., 2012). Furthermore, depending on the tissue environment, human macrophages are polarized toward either M1 or M2 subsets which display different functionality (Biswas and Mantovani, 2010). LPS stimulation induces a change of unpolarized (M0) and M2 macrophages toward an M1 phenotype and then leads to a strong activation of autologous NK cells (Bellora et al., 2010). Furthermore, human cytomegalovirus infected M1 macrophages secrete IL-6, IL-12, TNF-α, and IFN-γ whereas M2 macrophages produce only low levels of TNF-α, IFN-γ, and IL-10 (Romo et al., 2011) The emerging complexity of interactions between NK cells and macrophages makes the understanding of the pathways implicated *in vivo* in steady state or during infection an ongoing challenge.

## Concluding remarks

Activation of NK cells by many pathogens is triggered in part by signals coming from macrophages. The precise molecular interactions that underlie macrophages and NK cell communication need to be further investigated. It will be interesting to better define which implications this crosstalk might have on the adaptive immune response regarding the complex interactions of the different partners involved.

### Conflict of interest statement

The authors declare that the research was conducted in the absence of any commercial or financial relationships that could be construed as a potential conflict of interest.

## References

[B1] Aranha F. C. Ribeiro U. Jr. Basse P. Corbett C. E. Laurenti M. D. (2005). Interleukin-2-activated natural killer cells may have a direct role in the control of Leishmania (Leishmania) amazonensis promastigote and macrophage infection. Scand. J. Immunol. 62, 334–341 10.1111/j.1365-3083.2005.01681.x 16253120

[B2] Atochina O. Harn D. (2005). LNFPIII/LeX-stimulated macrophages activate natural killer cells via CD40-CD40L interaction. Clin. Diagn. Lab. Immunol. 12, 1041–1049 10.1128/CDLI.12.9.1041-1049.2005 16148169 PMC1235802

[B3] Baratin M. Roetynck S. Lepolard C. Falk C. Sawadogo S. Uematsu S. (2005). Natural killer cell and macrophage cooperation in MyD88-dependent innate responses to *Plasmodium falciparum*. Proc. Natl. Acad. Sci. U.S.A. 102, 14747–14752 10.1073/pnas.0507355102 16203971 PMC1253601

[B4] Basu S. Eriksson M. Pioli P. A. Conejo-Garcia J. Mselle T. F. Yamamoto S. (2009). Human uterine NK cells interact with uterine macrophages via NKG2D upon stimulation with PAMPs. Am. J. Reprod. Immunol. 61, 52–61 10.1111/j.1600-0897.2008.00661.x 19086992 PMC2902174

[B5] Bellora F. Castriconi R. Dondero A. Reggiardo G. Moretta L. Mantovani A. (2010). The interaction of human natural killer cells with either unpolarized or polarized macrophages results in different functional outcomes. Proc. Natl. Acad. Sci. U.S.A. 107, 21659–21664 10.1073/pnas.1007654108 21118979 PMC3003022

[B6] Bellora F. Castriconi R. Doni A. Cantoni C. Moretta L. Mantovani A. (2012). M-CSF induces the expression of a membrane-bound form of IL-18 in a subset of human monocytes differentiating *in vitro* toward macrophages. Eur. J. Immunol. 42, 1618–1626 10.1002/eji.201142173 22678914

[B7] Biswas S. K. Mantovani A. (2010). Macrophage plasticity and interaction with lymphocyte subsets: cancer as a paradigm. Nat. Immunol. 11, 889–896 10.1038/ni.1937 20856220

[B8] Brill K. J. Li Q. Larkin R. Canaday D. H. Kaplan D. R. Boom W. H. (2001). Human natural killer cells mediate killing of intracellular *Mycobacterium tuberculosis* H37Rv via granule-independent mechanisms. Infect. Immun. 69, 1755–1765 10.1128/IAI.69.3.1755-1765.2001 11179353 PMC98082

[B9] Chace J. H. Hooker N. A. Mildenstein K. L. Krieg A. M. Cowdery J. S. (1997). Bacterial DNA-induced NK cell IFN-gamma production is dependent on macrophage secretion of IL-12. Clin. Immunol. Immunopathol. 84, 185–193 10.1006/clin.1997.4380 9245551

[B10] Coudert J. D. Zimmer J. Tomasello E. Cebecauer M. Colonna M. Vivier E. (2005). Altered NKG2D function in NK cells induced by chronic exposure to NKG2D ligand-expressing tumor cells. Blood 106, 1711–1717 10.1182/blood-2005-03-0918 15886320

[B11] Dalbeth N. Gundle R. Davies R. J. Lee Y. C. McMichael A. J. Callan M. F. (2004). CD56bright NK cells are enriched at inflammatory sites and can engage with monocytes in a reciprocal program of activation. J. Immunol. 173, 6418–6426 15528382 10.4049/jimmunol.173.10.6418

[B12] Denis M. Keen D. L. Parlane N. A. Storset A. K. Buddle B. M. (2007). Bovine natural killer cells restrict the replication of *Mycobacterium bovis* in bovine macrophages and enhance IL-12 release by infected macrophages. Tuberculosis (Edinb.) 87, 53–62 10.1016/j.tube.2006.03.005 16730232

[B13] Elhaik-Goldman S. Kafka D. Yossef R. Hadad U. Elkabets M. Vallon-Eberhard A. (2011). The natural cytotoxicity receptor 1 contribution to early clearance of *Streptococcus pneumoniae* and to natural killer-macrophage cross talk. PLoS ONE 6:e23472 10.1371/journal.pone.0023472 21887255 PMC3161738

[B14] Haller D. Serrant P. Granato D. Schiffrin E. J. Blum S. (2002). Activation of human NK cells by staphylococci and lactobacilli requires cell contact-dependent costimulation by autologous monocytes. Clin. Diagn. Lab. Immunol. 9, 649–657 10.1128/CDLI.9.3.649-657.2002 11986274 PMC119993

[B15] Hamerman J. A. Ogasawara K. Lanier L. L. (2004). Cutting edge: toll-like receptor signaling in macrophages induces ligands for the NKG2D receptor. J. Immunol. 172, 2001–2005 14764662 10.4049/jimmunol.172.4.2001

[B16] Horowitz A. Stegmann K. A. Riley E. M. (2011). Activation of natural killer cells during microbial infections. Front. Immun. 2:88 10.3389/fimmu.2011.00088 22566877 PMC3342047

[B17] Hou X. Zhou R. Wei H. Sun R. Tian Z. (2009). NKG2D-retinoic acid early inducible-1 recognition between natural killer cells and Kupffer cells in a novel murine natural killer cell-dependent fulminant hepatitis. Hepatology 49, 940–949 10.1002/hep.22725 19177594

[B18] Kim E. O. Kim N. Kim T. J. Kim K. Kim T. W. Kumar V. (2010). Unidirectional signaling triggered through 2B4 (CD244), not CD48, in murine NK cells. J. Leukoc. Biol. 88, 707–714 10.1189/jlb.0410198 20647560

[B19] Klezovich-Benard M. Corre J. P. Jusforgues-Saklani H. Fiole D. Burjek N. Tournier J. N. (2012). Mechanisms of NK cell-macrophage *Bacillus anthracis* crosstalk: a balance between stimulation by spores and differential disruption by toxins. PLoS Pathog. 8:e1002481 10.1371/journal.ppat.1002481 22253596 PMC3257302

[B20] Kloss M. Decker P. Baltz K. M. Baessler T. Jung G. Rammensee H. G. (2008). Interaction of monocytes with NK cells upon Toll-like receptor-induced expression of the NKG2D ligand MICA. J. Immunol. 181, 6711–6719 18981088 10.4049/jimmunol.181.10.6711

[B21] Lapaque N. Walzer T. Meresse S. Vivier E. Trowsdale J. (2009). Interactions between human NK cells and macrophages in response to Salmonella infection. J. Immunol. 182, 4339–4348 10.4049/jimmunol.0803329 19299734

[B22] Lauzon W. Lemaire I. (1994). Alveolar macrophage inhibition of lung-associated NK activity: involvement of prostaglandins and transforming growth factor-beta 1. Exp. Lung Res. 20, 331–349 7988495 10.3109/01902149409064391

[B23] Lemaire I. St-Jean M. (1990). Modulation of lung-associated natural killer activity by resident and activated alveolar macrophages. Immunol. Invest. 19, 27–40 2338360 10.3109/08820139009042023

[B24] Lunemann A. Lunemann J. D. Roberts S. Messmer B. Barreira da Silva R. Raine C. S. (2008). Human NK cells kill resting but not activated microglia via NKG2D- and NKp46-mediated recognition. J. Immunol. 181, 6170–6177 18941207 10.4049/jimmunol.181.9.6170 PMC2596922

[B25] Mathew S. O. Rao K. K. Kim J. R. Bambard N. D. Mathew P. A. (2009). Functional role of human NK cell receptor 2B4 (CD244) isoforms. Eur. J. Immunol. 39, 1632–1641 10.1002/eji.200838733 19499526

[B25a] Michel T. Poli A. Domingues O. Mauffray M. Theresine M. Brons N. H. (2012). Mouse lung and spleen natural killer cells have phenotypic and functional differences, in part influenced by macrophages. PLoS ONE 7:e51230 10.1371/journal.pone.0051230 23227255 PMC3515449

[B26] Nausch N. Galani I. E. Schlecker E. Cerwenka A. (2008). Mononuclear myeloid-derived “suppressor” cells express RAE-1 and activate natural killer cells. Blood 112, 4080–4089 10.1182/blood-2008-03-143776 18753637 PMC2582006

[B27] Nedvetzki S. Sowinski S. Eagle R. A. Harris J. Vely F. Pende D. (2007). Reciprocal regulation of human natural killer cells and macrophages associated with distinct immune synapses. Blood 109, 3776–3785 10.1182/blood-2006-10-052977 17218381

[B28] Newman K. C. Riley E. M. (2007). Whatever turns you on: accessory-cell-dependent activation of NK cells by pathogens. Nat. Rev. Immunol. 7, 279–291 10.1038/nri2057 17380157

[B29] Ogasawara K. Hamerman J. A. Hsin H. Chikuma S. Bour-Jordan H. Chen T. (2003). Impairment of NK cell function by NKG2D modulation in NOD mice. Immunity 18, 41–51 10.1016/S1074-7613(02)00505-8 12530974

[B30] Raz E. (2007). Organ-specific regulation of innate immunity. Nat. Immunol. 8, 3–4 10.1038/ni0107-3 17179960

[B31] Richards D. M. Hettinger J. Feuerer M. (2012). Monocytes and macrophages in cancer: development and functions. Cancer Microenviron. [Epub ahead of print]. 10.1007/s12307-012-0123-x 23179263 PMC3717063

[B32] Romo N. Magri G. Muntasell A. Heredia G. Baia D. Angulo A. (2011). Natural killer cell-mediated response to human cytomegalovirus-infected macrophages is modulated by their functional polarization. J. Leukoc. Biol. 90, 717–726 10.1189/jlb.0311171 21742939

[B33] Schierloh P. Aleman M. Yokobori N. Alves L. Roldan N. Abbate E. (2005). NK cell activity in tuberculosis is associated with impaired CD11a and ICAM-1 expression: a regulatory role of monocytes in NK activation. Immunology 116, 541–552 10.1111/j.1365-2567.2005.02259.x 16313368 PMC1802446

[B34] Scott M. J. Hoth J. J. Stagner M. K. Gardner S. A. Peyton J. C. Cheadle W. G. (2004). CD40-CD154 interactions between macrophages and natural killer cells during sepsis are critical for macrophage activation and are not interferon gamma dependent. Clin. Exp. Immunol. 137, 469–477 10.1111/j.1365-2249.2004.02547.x 15320895 PMC1809143

[B35] Siren J. Sareneva T. Pirhonen J. Strengell M. Veckman V. Julkunen I. (2004). Cytokine and contact-dependent activation of natural killer cells by influenza A or Sendai virus-infected macrophages. J. Gen. Virol. 85(Pt 8), 2357–2364 10.1099/vir.0.80105-0 15269377

[B36] Small C. L. McCormick S. Gill N. Kugathasan K. Santosuosso M. Donaldson N. (2008). NK cells play a critical protective role in host defense against acute extracellular *Staphylococcus aureus* bacterial infection in the lung. J. Immunol. 180, 5558–5568 18390740 10.4049/jimmunol.180.8.5558

[B37] Soudja S. M. Ruiz A. L. Marie J. C. Lauvau G. (2012). Inflammatory monocytes activate memory CD8(+) T and innate NK lymphocytes independent of cognate antigen during microbial pathogen invasion. Immunity 37, 549–562 10.1016/j.immuni.2012.05.029 22940097 PMC3456987

[B38] Souza-Fonseca-Guimaraes F. Adib-Conquy M. Cavaillon J. M. (2012). Natural killer (NK) cells in antibacterial innate immunity: angels or devils? Mol. Med. 18, 270–285 10.2119/molmed.2011.00201 22105606 PMC3324953

[B39] Takayama T. Kamada N. Chinen H. Okamoto S. Kitazume M. T. Chang J. (2010). Imbalance of NKp44(+)NKp46(-) and NKp44(-)NKp46(+) natural killer cells in the intestinal mucosa of patients with Crohn's disease. Gastroenterology 139, 882–892, 892.e881–892.e883. 10.1053/j.gastro.2010.05.040 20638936

[B40] Taylor P. R. Martinez-Pomares L. Stacey M. Lin H. H. Brown G. D. Gordon S. (2005). Macrophage receptors and immune recognition. Annu. Rev. Immunol. 23, 901–944 10.1146/annurev.immunol.23.021704.115816 15771589

[B41] Tripp C. S. Wolf S. F. Unanue E. R. (1993). Interleukin 12 and tumor necrosis factor alpha are costimulators of interferon gamma production by natural killer cells in severe combined immunodeficiency mice with listeriosis, and interleukin 10 is a physiologic antagonist. Proc. Natl. Acad. Sci. U.S.A. 90, 3725–3729 10.1073/pnas.90.8.3725 8097322 PMC46374

[B42] Tu Z. Bozorgzadeh A. Pierce R. H. Kurtis J. Crispe I. N. Orloff M. S. (2008). TLR-dependent cross talk between human Kupffer cells and NK cells. J. Exp. Med. 205, 233–244 10.1084/jem.20072195 18195076 PMC2234385

[B43] Vankayalapati R. Garg A. Porgador A. Griffith D. E. Klucar P. Safi H. (2005). Role of NK cell-activating receptors and their ligands in the lysis of mononuclear phagocytes infected with an intracellular bacterium. J. Immunol. 175, 4611–4617 16177106 10.4049/jimmunol.175.7.4611

[B44] Welte S. Kuttruff S. Waldhauer I. Steinle A. (2006). Mutual activation of natural killer cells and monocytes mediated by NKp80-AICL interaction. Nat. Immunol. 7, 1334–1342 10.1038/ni1402 17057721

[B45] Zhou Z. Zhang C. Zhang J. Tian Z. (2012). Macrophages help NK cells to attack tumor cells by stimulatory NKG2D ligand but protect themselves from NK killing by inhibitory ligand Qa-1. PLoS ONE 7:e36928 10.1371/journal.pone.0036928 22629344 PMC3356357

[B46] Zoller M. Matzku S. (1982). Rat macrophages inhibit natural killer (NK) cell activity against adherent growing target cells. Immunobiology 163, 497–510 10.1016/S0171-2985(82)80063-6 6984420

